# Polyphenols from the mangosteen (Garcinia mangostana) fruit for breast and prostate cancer

**DOI:** 10.3389/fphar.2013.00080

**Published:** 2013-06-26

**Authors:** Gongbo Li, Stacey Thomas, Jeremy J. Johnson

**Affiliations:** ^1^Department of Pharmacy Practice, College of Pharmacy, University of Illinois at ChicagoChicago, IL, USA; ^2^Carcinogenesis and Chemoprevention Research Program, University of Illinois Cancer CenterChicago, IL, USA

**Keywords:** α-Mangostin, mangosteen, prostate cancer, extract, xanthone

## Abstract

The mangosteen (*Garcinia mangostana*) is a tropical fruit native to Southeast Asia and has long been reported to contain multiple health promoting properties. This fruit is an abundant source of xanthones, a class of polyphenolic compounds with a distinctive tricyclic aromatic ring system and is largely responsible for its biological activities including anti-cancer activity. Herein we describe the anti-cancer activity and mechanisms of mangosteen polyphenolic xanthones including α-Mangostin against breast cancer and prostate cancer. So far, extracts and individual xanthones have been found to induce apoptosis and inhibit proliferation on cancer cells *in vitro* and *in vivo*. Based on the reported findings there is clear evidence that these polyphenols target multiple signaling pathways involved in cell cycle modulation and apoptosis. Further work is required to understand its potential for health promotion and potential drug discovery for prostate and breast cancer chemoprevention.

## THE MANGOSTEEN FRUIT

The mangosteen (*Garcinia mangostana*) is a tropical fruit native to Southeast Asia that has long been noted for its medicinal and health promoting properties (Obolskiy et al., 2009). The mangosteen tree is a slow growing plant which ranges from 6 to 25 m in height and produces flowers that are red and green in color and are roughly 4–5 cm in size (Obolskiy et al., 2009). The exocarp of the fruit is deep purple in color with a white pulp and typically contains 6–8 arils (Chin and Kinghorn, 2008). The fruit has been used for several hundred years in Southeast Asia for the treatment of wounds, inflammation, and a variety of infections (Chin and Kinghorn, 2008).

## DIETARY XANTHONES, A SOURCE OF POLYPHENOLS FROM THE MANGOSTEEN FRUIT

Recently, the mangosteen has been noted to be an abundant source of a class of polyphenols known as xanthones. The diverse structure and chemical properties of xanthones have been reported to have a variety of health promoting properties including anti-inflammatory, anti-oxidant, and anti-cancer activity. The chemical structure of a xanthone consists of a three ring system which contains several different functional groups including isoprene, methoxy, and phenyl groups as well as aromatic protons, phenolic hydroxyl groups, hydroxyl protons, and dihydrofuran rings (Shan et al., 2011). Xanthones can be classified into several groups including simple oxygenated xanthones, prenylated xanthones, xanthone glycosides, and xanthonolignoids based on these substituents.

To date, more than 60 different xanthones have been isolated from the mangosteen pericarp, bark, and roots including α-Mangostin, γ-Mangostin, gartanin, 8-deoxygartanin, and 9-hydroxycalabaxanthone (Obolskiy et al., 2009). A wide variety of pharmacological activities have been associated with the fruit and more specifically the individual xanthones therein have been shown to target multiple signaling pathways. Among the xanthones, α-Mangostin is the most abundant and is also one of the most studied xanthones due to its potential therapeutic properties.

Due to the increasing incidence of cancer, there has been a growing interest in the management of the disease through dietary chemoprevention, which is the use of naturally occurring phytochemicals to slow down the process of carcinogenesis. Xanthones, with their unique physical and chemical properties, are a promising candidate in the study of dietary chemoprevention due to their health promoting properties as well as their apparent safety. It has long been suggested that many xanthones from the mangosteen fruit, including α-Mangostin, possess anti-cancer properties including initiation of apoptosis through the regulation of cell death pathways, the suppression of cancer cell proliferation and metastasis through inhibition of anti-apoptotic molecules, and cell cycle arrest (Shan et al., 2011). They have also been observed to inhibit the initiation, promotion, and progression of carcinogenesis in several animal models. Xanthones have been shown to modulate cell signaling pathways that are deregulated in cancer cells.

In general, xanthone anti-cancer activity has been shown to decrease with the addition of hydroxyl groups on the 5-carbon sidechain while the highest anti-cancer activity is seen xanthones containing tetraoxygen groups with two 5-carbon isoprenyl groups in rings A and B (Obolskiy et al., 2009; Shan et al., 2011). Of all the xanthones, α-Mangostin has demonstrated the greatest anti-cancer activity in prostate, breast, lung, and colorectal cancer (Shan et al., 2011).

In recent years, there has been growing interest in the ability of natural compounds such as α-Mangostin to serve as chemopreventive agents in the treatment of prostate and breast cancer. In most cases the progression of prostate and breast cancer occurs very slowly, spanning over a period of many years, with very distinct stages of initiation, promotion, and progression. Aside from superficial cancers of the skin, prostate cancer is the second leading cause of cancer-related death in the United States (Arcangeli et al., 2012). Annually, about 660,000 men worldwide are diagnosed with prostate cancer. The disease typically affects males over the age of 50 with 80% of cases occurring in men over the age of 65. In 2010, nearly 1.5 million people were diagnosed with breast cancer worldwide. In 2011, an estimated 230,480 new cases of invasive breast cancer will be diagnosed among women in the United States, as well as an estimated 57,650 additional cases of *in situ* breast cancer according to American Cancer Society.

As a result of its long latency, even a small delay in carcinogenesis can set back the onset of disease and improve quality of life. It has been reported that less than 10% of prostate and breast cancer cases are inherited which suggests that a combination of genetics, environment, and lifestyle choices may help initiate the development of the disease (Carter et al., 1992). Several migratory studies have demonstrated a link between natural dietary agents and a lower overall incidence of prostate cancer (Khan and Mukhtar, 2012). Generally, the incidence of prostate cancer is significantly lower in countries where people consume low-fat, plant-based diets (Arcangeli et al., 2012). This has led investigators to suggest that the role of the diet is an important factor in contributing to worldwide cancer incidence.

The lethality of prostate cancer originates from several factors including high oxidative stress of cancer cells and leading to the deregulation of multiple cellular signaling pathways including those responsible for regulating cell proliferation and the initiation of apoptosis (Johnson et al., 2008). Due to its high incidence, long latency, and link to diet and lifestyle, chemoprevention through the use of natural dietary compounds such as α-Mangostin may be an effective method in the management of breast and prostate cancer.

## ANTI-CANCER ACTIVITY OF MANGOSTEEN EXTRACT TOWARD BREAST CANCER

In several studies, crude extracts from pericarp of mangosteen were evaluated for their cytotoxic activity against human breast adenocarcinoma cell line. In one study (Moongkarndi et al., 2004a), a crude methanolic extract (CME) from mangosteen pericarp was found to significantly inhibit the proliferation of human breast cancer cell line SKBR3 in a dose-dependent manner with an ED_50_ of 9.25 ± 0.64μg/ml. CME also induced apoptosis on SKBR3 cells with the findings of morphological changes and oligonucleosomal DNA fragments. In a second study (Moongkarndi et al., 2004b), an ethanolic extract from mangosteen pericarp was found to have anti-proliferative activity on SKBR3 cells with an IC_50_ of 15.45 ± 0.50 μg/ml. Based on these observations investigators have begun to evaluate individual xanthones for their anti-cancer potential in both breast and prostate cancer.

## ANTI-CANCER ACTIVITY OF INDIVIDUAL XANTHONES TOWARD BREAST CANCER

Suksamrarn et al. (2006) evaluated the *in vitro* cytotoxicity of 19 xanthones from the young fruit (7-week maturity stage) of mangosteen against different cell lines from human breast cancer (BC-1) epidermoid carcinoma of the mouth (KB), and small cell lung cancer (NCI-H187). In BC-1 cells, α-Mangostin was found to be the most potent with an IC_50_ value of 0.92 μg/ml (i.e., 2.24 μM) followed by garcinone E and γ-Mangostin with IC_50_ values of 1.44 and 1.6 μg/ml, respectively. In total, 13 of 19 xanthones were observed to decrease cell viability at or below 20 μg/ml (Suksamrarn et al., 2006).

Kurose et al. (2012) evaluated α-Mangostin in the human breast cancer cell line MDA-MB231 observing evidence of apoptosis by the findings of significantly elevated ssDNA level and caspase 3, 8, and 9 levels. Significant cytochrome c release was also observed in α-Mangostin-treated cells, suggesting α-Mangostin induced MDA-MB231 cell apoptosis through the mitochondrial pathway. α-Mangostin treatment also induced cell cycle arrest through upregulation of the cyclin-dependent kinase (CDK) inhibitor p21^cip1^ and cell cycle checkpoint regulator CHEK2 (Kurose et al., 2012).

Shibata et al. (2011) evaluated the anti-breast cancer activity of α-Mangostin on a xenograft metastatic mammary cancer mice model. In this study, BJMC3879luc2 tumor cells were subcutaneously implanted in BALB/c mice. After 3 weeks mini osmotic pumps were implanted and set to deliver 20 mg/kg/day of α-Mangostin for a total of 6 weeks. The results indicated that the α-Mangostin-treated group had a significantly higher survival rate as well as smaller average tumor volume (785 ± 170 and 744 ± 292 mm^3^ for 10 and 20 mg/kg/day α-Mangostin, respectively vs. 993 ± 612 mm^3^ for the control). This represents a 21 and 25% smaller tumor in the α-Mangostin treatment groups. In addition, fewer lymph nodes metastases were observed in treated mice compared to controls.The authors also observed an increase of cells in the G1-phase, a decrease of cells in the S-phase and mitochondria-mediated apoptosis after treating tumor cells with α-Mangostin *in vitro*. In addition, they found that α-Mangostin significantly decreased the oncogenic phospho-Akt-threonine 308 expression in both treated breast cancer cells *in vitro* and breast cancer tissues *in vivo*.

Jung et al. (2006) evaluated α-Mangostin and γ-Mangostin for their anti-cancer activity against preneoplastic lesions induced by 7,12-dimethylbenz[α]anthracene (DMBA), a powerful organ-specific carcinogen, in a mouse mammary organ culture (MMOC) system. Briefly, mice were pretreated for 9 days with estradiol and progesterone, sacrificed and mammary glands dissected and subjected to culture for 10 days under appropriate conditions. Glands were exposed to 2 μg/mL DMBA for 24 h then permitted to regress by withdrawing all hormones except insulin for 14 additional days. Xanthones were present in the medium during days 1–10 of culture; mammary glands were scored for the incidence of lesions. At a concentration of 10 μg/mL (i.e., 24.36 and 25.23 μM for α-Mangostin and γ-Mangostin, respectively), the percent inhibitions of α-Mangostin and γ-Mangostin were 57.1 and 42.9, respectively. α-Mangostin was then further determined to have an IC_50_ of 1.0 μg/mL (2.44 μM) in a dose–response MMOC assay.

Balunas et al. evaluated the aromatase (estrogen synthase) inhibitory activity of 12 xanthones from mangosteen pericarp (Balunas et al., 2008). Garcinone D, garcinone E, α-Mangostin, and γ-Mangostin were found to have inhibitory activity using a cell free, enzyme-based microsomal aromatase inhibition assay. Following the cell-based assay, SKBR3 breast cancer cells which are known to express high levels of aromatase were evaluated to determine if these observations will occur in a cell-based system. γ-Mangostin was found to be the most potent aromatase inhibitor, followed by garcinone D, α-Mangostin and garcinone E.

## ANTI-CANCER ACTIVITY OF INDIVIDUAL XANTHONES TOWARD PROSTATE CANCER

Hung et al. (2009) evaluated the anti-metastatic effect of α-Mangostin in the human prostate carcinoma cell line PC-3, which originated from bone metastasis. α-Mangostin was found to inhibit the adhesion, migration, and invasion of PC-3 cells by respective *in vitro* assays. Further mechanism investigation found that α-Mangostin significantly decreased the expressions of multiple matrix degrading proteinases, including matrix metalloproteinase-2 (MMP-2), matrix metalloproteinase-9 (MMP-9), and urokinase-plasminogen activator (u-PA), which are all required by cancer cell metastasis process. α-Mangostin also inhibited the phosphorylation of c-Jun N-terminal kinase 1 and 2 (JNK1/2) as well as the activation of nuclear factor kappa B (NF-κB), oncogene c-Fos and c-Jun, which are all associated with invasion and metastasis of cancer cells. Similar results were also found when human melanoma cell line SK-MEL-28 and squamous cell carcinoma cell line A-431 were treated with α-Mangostin (Wang et al., 2012).

Han et al. (2009) evaluated the anti-cancer activity of α-Mangostin on androgen-dependent/androgen responsive human prostate cancer cell LNCaP in an *in vivo* hollow fiber model. In brief, LNCaP cells were infused into polyvinylidene fluoride hollow fibers. The fibers were then heat sealed at 2 cm intervals and cut in the middle of seals to generate fibers. Fibers were cultured overnight, and one set of them is evaluated for viable cell by 3-(4, 5-dimethylthiazol-2-yl)-2, 5-diphenyltetrazolium bromide (MTT) assay. Another set of fibers were intraperitoneally implanted into NCr *nu/nu* mice (designated day 0). Mice then received four daily intraperitoneal injections of α-Mangostin on days 3, 4, 5, and 6. On day 7, mice were sacrificed and fibers were retrieved then placed into 6-well plates, where viable cell mass contained within fibers were determined with MTT assay. Interestingly, α-Mangostin did not exhibit cytotoxic activity against LNCaP cells in the hollow fiber model at a range of doses from 2.5 mg/kg up to 20 mg/kg. Possible explanation is that the 7-day study is too short for α-Mangostin to show efficacy or 20 mg/kg of α-Mangostin is not enough to achieve an observable cytotoxicity on LNCaP cells in the specific model.

Recently, we evaluated the anti-cancer activity of α-Mangostin on several human prostate cancer cell lines as well as a xenograft mouse model (Johnson et al., 2012). α-Mangostin was found to have significantly lower IC_50_ values on all four prostate cancer cell lines (LNCaP, 22Rv1, DU145, and PC-3) compared to epigallocatechin 3-gallate (EGCG), genistein, and 3,3′-diindolylmethane (DIM). The IC_50_ of α-Mangostin was 5.9, 6.9, 22.5, and 12.7 μM for LNCaP, 22Rv1, DU145, and PC3, respectively. Interestingly, when human normal prostate epithelial cells (PrECs) were treated with 15 μM of α-Mangostin there was no observable inhibition on cell growth compared to control cells.In prostate cancer cells α-Mangostin was also found to inhibit colony formation and induce G1 cell cycle arrest. Further mechanism investigation found that 10 μM α-Mangostin significantly inhibited the kinase activity of cyclinA1/CDK2 (34.2% ± 0.1), cyclinA2/CDK2 (13.2% ± 0.4), cyclinD1/CDK4 (59.2 ± 1.4), and cyclinD3/CDK6 (5.5% ± 0.3). No direct inhibition of kinase activity was observed on JNK1 or JNK2 at 1 or 10 μM α-Mangostin which is inconsistent with the above-mentioned study for unknown reason. When we detected the possible expression changes of proteins upstream of CDK4 that included the INK proteins, p27 Kip1 and CDK4 in 22Rv1 cells, we found an increase in p27 Kip1. This was in agreement with a previous study of α-Mangostin on human colon cancer DLD-1 cells (Matsumoto et al., 2005) and p27 overexpression has been reported to induce apoptosis in several different cancer cell lines (Katayose et al., 1997; Wang et al., 1997). Further, the previous study found that α-Mangostin downregulated the expression of cyclinA and cyclinD1, which is also in concert with our kinase inhibition results. In addition, a dose-dependent decrease in the downstream targets of CDK4, specifically the phosphorylation sites of retinoblastoma protein at residues Ser780, Ser795, and Ser807/811, was observed. Based on these results and molecular modeling, we generated an α-Mangostin/CDK4 binding hypothesis. Our data provided a more thorough molecular understanding to previous reports of α-Mangostin decreasing cell viability and promoting G1 cell cycle arrest (Matsumoto et al., 2003, 2004, 2005). Next, mice were subcutaneously implanted with 22Rv1 cells followed and treated with α-Mangostin (100 mg/kg orally) resulting in a 65% smaller tumor in the treatment group.

## CONCLUSION

In recent years, the mangosteen has become a popular dietary supplement for its potential health promoting properties. The mangosteen fruit is unique in that it is the most abundant source of a diverse “natural” chemical library of xanthones and is largely responsible for their health promoting properties. In light of the interesting observations of these xanthones to modulate multiple signaling pathways it is clear that further work is required to understand its potential for health promotion and potential drug discovery. This is especially true in that the mangosteen fruit is becoming a popular dietary supplement and juice in recent years.

## Conflict of Interest Statement

The authors declare that the research was conducted in the absence of any commercial or financial relationships that could be construed as a potential conflict of interest.

## References

[B1] Arcangeli S. Pinzi V. Arcangeli G. (2012). Epidemiology of prostate cancer and treatment remarks. *World J. Radiol.* 4 241–246 10.4329/wjr.v4.i6.241 22778875 PMC3391668

[B2] Balunas M. J. Su B. Brueggemeier R. W. Kinghorn A. D. (2008). Xanthones from the botanical dietary supplement mangosteen (*Garcinia mangostana*) with aromatase inhibitory activity. *J. Nat. Prod.* 71 1161–1166 10.1021/np8000255 18558747 PMC2572570

[B3] Carter B. S. Beaty T. H. Steinberg G. D. Childs B. Walsh P. C. (1992). Mendelian inheritance of familial prostate cancer. *Proc. Natl. Acad. Sci. U.S.A.* 89 3367–3371 10.1073/pnas.89.8.3367 1565627 PMC48868

[B4] Chin Y. W. Kinghorn A. D. (2008). Structural characterization, biological effects, and synthetic studies on xanthones from mangosteen (*Garcinia mangostana*), a popular botanical dietary supplement. *Mini Rev. Org. Chem.* 5 355–364 10.2174/157019308786242223 21562610 PMC3090081

[B5] Han A. R. Kim J. A. Lantvit D. D. Kardono L. B. Riswan S. Chai H. (2009). Cytotoxic xanthone constituents of the stem bark of *Garcinia mangostana* (mangosteen). *J. Nat. Prod.* 72 2028–2031 10.1021/np900517h 19839614 PMC2887388

[B6] Hung S. H. Shen K. H. Wu C. H. Liu C. L. Shih Y. W. (2009). Alpha-mangostin suppresses PC-3 human prostate carcinoma cell metastasis by inhibiting matrix metalloproteinase-2/9 and urokinase-plasminogen expression through the JNK signaling pathway. *J. Agric. Food Chem.* 57 1291–1298 10.1021/jf8032683 19178296

[B7] Johnson J. J. Petiwala S. M. Syed D. N. Rasmussen J. T. Adhami V. M. Siddiqui I. A. (2012). Alpha-mangostin, a xanthone from mangosteen fruit, promotes cell cycle arrest in prostate cancer and decreases xenograft tumor growth. *Carcinogenesis* 33 413–419 10.1093/carcin/bgr291 22159229 PMC3271271

[B8] Johnson J. J. Syed D. N. Heren C. R. Suh Y. Adhami V. M. Mukhtar H. (2008). Carnosol, a dietary diterpene, displays growth inhibitory effects in human prostate cancer PC3 cells leading to G2-phase cell cycle arrest and targets the 5^′^-AMP-activated protein kinase (AMPK) pathway. *Pharm. Res.* 25 2125–2134 10.1007/s11095-008-9552-0 18286356 PMC2994272

[B9] Jung H. A. Su B. N. Keller W. J. Mehta R. G. Kinghorn A. D. (2006). Antioxidant xanthones from the pericarp of *Garcinia mangostana* (Mangosteen). *J. Agric. Food Chem.* 54 2077–2082 10.1021/jf052649z 16536578

[B10] Katayose Y. Kim M. Rakkar A. N. Li Z. Cowan K. H. Seth P. (1997). Promoting apoptosis: a novel activity associated with the cyclin-dependent kinase inhibitor p27. *Cancer Res.* 57 5441–5445 9407946

[B11] Khan N. Mukhtar H. (2012). Modulation of signaling pathways in prostate cancer by green tea polyphenols. *Biochem. Pharmacol*. 85 667–672 10.1016/j.bcp.2012.09.027 23041649 PMC3815652

[B12] Kurose H. Shibata M. A. Iinuma M. Otsuki Y. (2012). Alterations in cell cycle and induction of apoptotic cell death in breast cancer cells treated with alpha-mangostin extracted from mangosteen pericarp. *J. Biomed. Biotechnol.* 2012 672428 10.1155/2012/672428 PMC3332218 22577295

[B13] Matsumoto K. Akao Y. Kobayashi E. Ohguchi K. Ito T. Tanaka T. (2003). Induction of apoptosis by xanthones from mangosteen in human leukemia cell lines. *J. Nat. Prod.* 66 1124–1127 10.1021/np020546u 12932141

[B14] Matsumoto K. Akao Y. Ohguchi K. Ito T. Tanaka T. Iinuma M. (2005). Xanthones induce cell-cycle arrest and apoptosis in human colon cancer DLD-1 cells. *Bioorg. Med. Chem.* 13 6064–6069 10.1016/j.bmc.2005.06.065 16112579

[B15] Matsumoto K. Akao Y. Yi H. Ohguchi K. Ito T. Tanaka T. (2004). Preferential target is mitochondria in alpha-mangostin-induced apoptosis in human leukemia HL60 cells. *Bioorg. Med. Chem.* 12 5799–5806 10.1016/j.bmc.2004.08.034 15498656

[B16] Moongkarndi P. Kosem N. Kaslungka S. Luanratana O. Pongpan N. Neungton N. (2004a). Antiproliferation, antioxidation and induction of apoptosis by *Garcinia mangostana* (mangosteen) on SKBR3 human breast cancer cell line. *J. Ethnopharmacol.* 90 161–166 10.1016/j.jep.2003.09.048 14698525

[B17] Moongkarndi P. Kosem N. Luanratana O. Jongsomboonkusol S. Pongpan N. (2004b). Antiproliferative activity of Thai medicinal plant extracts on human breast adenocarcinoma cell line. *Fitoterapia* 75 375–377 10.1016/j.fitote.2004.01.010 15158999

[B18] Obolskiy D. Pischel I. Siriwatanametanon N. Heinrich M. (2009). *Garcinia mangostana* L.:* a phytochemical and pharmacological review*. *Phytother. Res.* 23 1047–1065 10.1002/ptr.2730 19172667

[B19] Shan T. Ma Q. Guo K. Liu J. Li W. Wang F. (2011). Xanthones from mangosteen extracts as natural chemopreventive agents: potential anticancer drugs. *Curr. Mol. Med.* 11 666–677 10.2174/156652411797536679 21902651 PMC3237908

[B20] Shibata M. A. Iinuma M. Morimoto J. Kurose H. Akamatsu K. Okuno Y. (2011). Alpha-Mangostin extracted from the pericarp of the mangosteen (*Garcinia mangostana* Linn) reduces tumor growth and lymph node metastasis in an immunocompetent xenograft model of metastatic mammary cancer carrying a p53 mutation. *BMC Med.* 9:69 10.1186/1741-7015-9-69 PMC3121600 21639868

[B21] Suksamrarn S. Komutiban O. Ratananukul P. Chimnoi N. Lartpornmatulee N. Suksamrarn A. (2006). Cytotoxic prenylated xanthones from the young fruit of *Garcinia mangostana*. *Chem. Pharm. Bull. (Tokyo)* 54 301–305 10.1248/cpb.54.301 16508181

[B22] Wang J. J. Sanderson B. J. Zhang W. (2012). Significant anti-invasive activities of alpha-mangostin from the mangosteen pericarp on two human skin cancer cell lines. *Anticancer Res.* 32 3805–3816 22993323

[B23] Wang X. Gorospe M. Huang Y. Holbrook N. J. (1997). p27Kip1 overexpression causes apoptotic death of mammalian cells. *Oncogene* 15 2991–2997 10.1038/sj.onc.1201450 9416843

